# Characterization of a Ruthenium(II) Complex in Singlet Oxygen-Mediated Photoelectrochemical Sensing

**DOI:** 10.1021/acs.langmuir.2c03042

**Published:** 2022-12-27

**Authors:** Margherita Verrucchi, Gina Elena Giacomazzo, Patrick Severin Sfragano, Serena Laschi, Luca Conti, Marco Pagliai, Cristina Gellini, Marilena Ricci, Enrico Ravera, Barbara Valtancoli, Claudia Giorgi, Ilaria Palchetti

**Affiliations:** † Dipartimento di Chimica Ugo Schiff, Università degli Studi di Firenze, Via della Lastruccia 3, 50019 Sesto Fiorentino (FI), Italy; ‡ CERM, Università degli Studi di Firenze, Via Luigi Sacconi 6, 50019 Sesto Fiorentino (FI), Italy; § CIRMMP, Via Luigi Sacconi 6, 50019 Sesto Fiorentino (FI), Italy

## Abstract

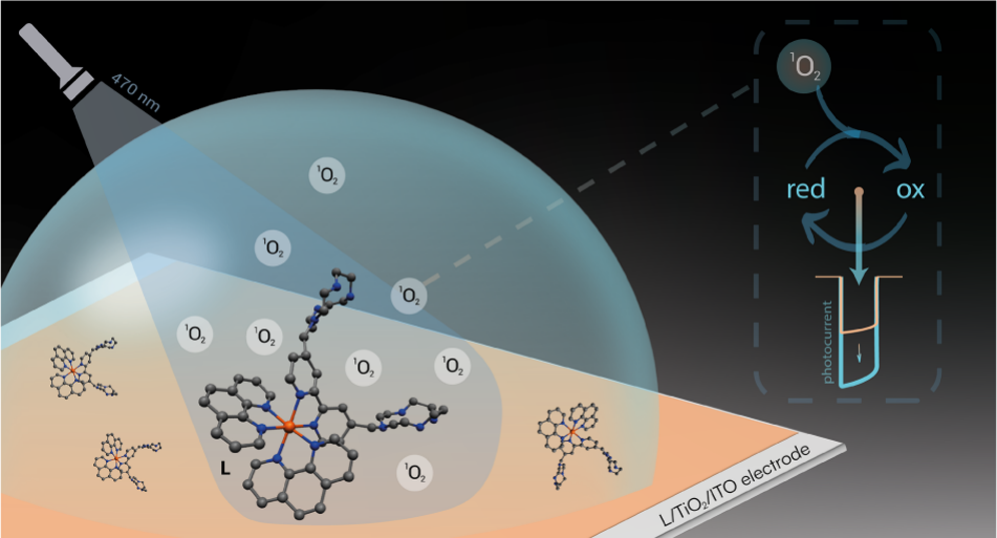

A water-soluble ruthenium(II) complex (L), capable of producing singlet oxygen (^1^O_2_) when irradiated with visible light, was used to modify the surface of an indium–tin oxide (ITO) electrode decorated with a nanostructured layer of TiO_2_ (TiO_2_/ITO). Singlet oxygen triggers the appearance of a cathodic photocurrent when the electrode is illuminated and biased at a proper reduction potential value. The L/TiO_2_/ITO electrode was first characterized with cyclic voltammetry, impedance spectroscopy, NMR, and Raman spectroscopy. The rate constant of singlet oxygen production was evaluated by spectrophotometric measurements. Taking advantage of the oxidative process initiated by ^1^O_2_, the analysis of phenolic compounds was accomplished. Particularly, the ^1^O_2_-driven oxidation of hydroquinone (HQ) produced quinone moieties, which could be reduced back at the electrode surface, biased at −0.3 V *vs* Ag/AgCl. Such a light-actuated redox cycle produced a photocurrent dependent on the concentration of HQ in solution, exhibiting a limit of detection (LOD) of 0.3 μmol dm^–3^. The L/TiO_2_/ITO platform was also evaluated for the analysis of *p*-aminophenol, a commonly used reagent in affinity sensing based on alkaline phosphatase.

## Introduction

Singlet oxygen (^1^O_2_) is considered a strong oxidation reagent with applications in the field of photooxidation of natural and manmade organic compounds,^1^ water treatment,^2^ and photodynamic therapy (PDT).^3^ Among the different applications, PDT is considered a promising cancer therapy, with ^1^O_2_ as one of the reactive oxygen species acting as a cytotoxic agent that damages cancer tissues.^4,5^ Recently, ^1^O_2_ was also successfully proposed as an *in situ*-generated reagent for photoelectrochemical (PEC) sensing approaches,^6,7^ under visible-light illumination. De Wael’s group, using air instead of added reactive reagents, was able to determine nanomolar levels of the antibiotic amoxicillin at a screen-printed TiO_2_ electrode modified with a fluorinated zinc phthalocyanine photosensitizer.^7^ The same detection scheme based on phthalocyanine-generated ^1^O_2_ was used to develop a PEC immunosensor for the determination of *Toxocara canis* antigens,^8^ with the *in situ*-generated ^1^O_2_ reacting with the enzymatically produced *p*-nitrophenol, the label of the affinity reaction.

Different photosensitizers have been used for *in situ*^1^O_2_ production and, among these, some complexes of Ru(II). These complexes show excited states with a pronounced triplet character due to the high spin–orbit coupling constant, which accelerates the rate of intersystem crossing from the singlet electronic excited state. A long lifetime of the triplet excited state of ruthenium(II) complexes allows the direct interaction between excited Ru(II) and ground-state molecular oxygen (^3^O_2_) and the production of highly reactive singlet oxygen (^1^O_2_). Thus, it is thermodynamically possible for many ruthenium(II) complexes to be very efficient singlet oxygen producers in aerated solutions.^3^

Recently, a ruthenium(II) complex [Ru(phen)2(*L’*)]^2+^ (Scheme S1 of the Supporting Information, where *phen* is for 1,10-phenanthroline and L’ is for 4,4′-bis-[methylen-(1,4,7,10-tetraazacyclododecane)]-2,2-bipyridine) has been recognized to be a good ^1^O_2_ producer.^9−11^ This compound looks interesting thanks to the peculiar polyazamacrocycle framework L’. In fact, the presence of this highly charged ligand confers on the metal complex a series of features, including the ability to effectively interact with important biological targets, such as DNA, and favors the interaction with the protein surface of hybrid Ru(II)–protein assemblies for therapeutic applications,^11^ including in PDT.^9^ Its good singlet oxygen-sensitizing properties make it a promising tool for the development of reagentless analytical approaches. The idea behind the design of such a ruthenium(II) complex is that the presence of charged polyamine frameworks appended to the metal-coordinated heteroaromatic units could confer to the resulting metal complexes better water solubility. In fact, the presence of ancillary ligands with extensive aromaticity useful for good singlet oxygen production gives molecular systems with low solubility in an aqueous medium. The presence of cyclen units can easily undergo protonation in an aqueous solution, making these systems soluble in water. Furthermore, these highly charged species could promote the physisorption of L on the electrode surface.

In this work, this novel [Ru(phen)_2_(L’)]^2+^ photosensitizer was used for PEC sensing. In particular, the complex was used for the determination of organic compounds such as phenols. To the best of our knowledge, singlet oxygen produced by the photoexcited ruthenium(II) complex has never been used for PEC sensing approaches. In this paper, an analytical procedure for the determination of phenols, such as hydroquinone (HQ), was developed.

**Scheme 1:**
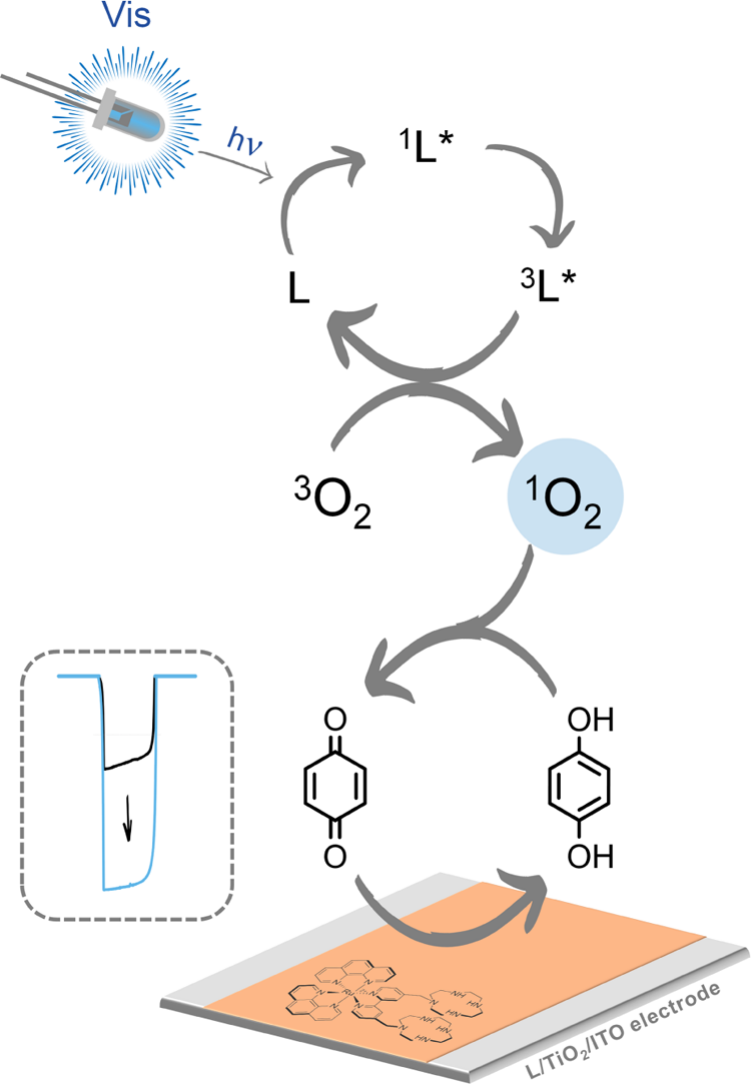
Scheme of the Reaction Mechanism Involving the Ruthenium(II) Complex L in the Detection of Quinones The ruthenium(II) complex is used to modify the surface of an indium–tin oxide (ITO) electrode decorated with a nanostructured layer of TiO_2_ (L/TiO_2_/ITO). Once irradiated with visible light, the complex produces singlet oxygen (^1^O_2_); this last oxidizes hydroquinone or *p*-aminophenol producing quinone moieties, which are reduced back at the electrode surface by applying a suitable working potential. The reduction photocurrent is then measured.

The proposed assay is based on the chemical reaction between *in situ*-generated singlet oxygen and HQ. In particular, HQ was chemically oxidized to the quinone form by the light-produced ^1^O_2_. The oxidized form is reduced back at the electrode surface, generating a measurable photocurrent proportional to HQ concentration (Scheme 1). In this way, without the addition of any external reagent, it is possible to perform measurements at potential values where interferences from electroactive molecules are less prone to contribute.

Furthermore, *p*-aminophenol (PAP) was also tested for its importance in many analytical applications^12^ and as a reaction product of alkaline phosphatase (AP) enzymatic hydrolysis of *p*-aminophenyl phosphate. AP is a widely used enzyme in optical and electrochemical biosensors as a label of an affinity reaction.^13−16^ Herein, we investigate the possibility of using AP as a label of affinity reactions in PEC biosensing at a TiO_2_/ITO electrode decorated with the ruthenium(II) photosensitizer L.

## Experimental Section

### Reagents

Water from a Milli-Q water purification system (Millipore, U.K.) was used to prepare the aqueous solutions. Potassium chloride (KCl), hydroquinone (C_6_H_6_O_2_), potassium hexacyanoferrate(II)/(III) (K_4_[Fe(CN)_6_]/K_3_[Fe(CN)_6_]), glycine, sodium dihydrogen phosphate (NaH_2_PO_4_·2H_2_O), sodium hydrogen phosphate (Na_2_HPO_4_·2H_2_O), streptavidin–alkaline phosphatase, acetic acid (CH_3_COOH), and sodium acetate anhydrous (CH_3_COONa) were purchased from Sigma (Milano, Italy). The dye, namely, [(phen)_2_Ru(4,4′-bisbipyciclen)]Cl_2_·5HCl (molecular weight: 11,685 g/mol), was synthesized following reference^17^ and kept refrigerated and away from light. Synthesis (Schemes S1 and S2) and characterization data (Figures S1 and S2) of the ruthenium complex can be found in the Supporting Information.

Screen-printed electrochemical cells (SPECs) were obtained from Ecobioservices and Researches SrL (EBSR), Sesto Fiorentino, Italy. The composition and dimensions have been reported elsewhere.^18^ SPECs were used as disposables without any previous cleaning treatment or modification.

### Electrode Fabrication

TiO_2_ electrodes were manufactured by doctor blading, coating a layer of nanocrystalline TiO_2_-based paste (18NR-T TiO_2_ Paste, Greatcell Solar, Elanora, Australia, former DyeSol) onto a clean ITO glass electrode. The square ITO-coated glass slides (70–100 Ω/sq) were obtained from Sigma, Milano, Italy. Before use, the ITO electrodes were gently polished with an EtOH-embedded cloth and finally rinsed with EtOH.

For the sintering step, the TiO_2_-coated electrodes were heated in an oven for 40 min at 500 °C to ensure complete combustion and removal of the organic content and thus enhance electron transport. Two layers were deposited; both after the first and the second layer, the electrode was heated in an oven for 40 min at 500 °C. Before use, the electrode was cooled down at room temperature.

### Electrode Modification

The nanocrystalline TiO_2_/ITO electrodes were incubated overnight (12 h) in a plastic reaction chamber containing 2 mL of 0.7 mmol dm^–3^ [(phen)_2_Ru(4,4′-bisbipyciclen)]Cl_2_·5HCl solution in Milli-Q water. After the coating, the samples were rinsed with water.

### Photoelectrochemical Measurements

Photoelectrochemical and electrochemical measurements were performed with an Autolab PGSTAT10 computerized electrochemical system equipped with the FRA2 frequency response analyzer and controlled by NOVA software (Metrohm). A Pt wire, a Ag/AgCl wire, and an L/TiO_2_/ITO electrode served as the auxiliary, pseudo-reference, and working electrodes, respectively. To overcome any problems related to the possible degradation of Ag/AgCl wire by light, all of the measurements carried out in this work are relativized with respect to their own blank, so any variation in stability of the pseudo-reference electrode is eliminated by data processing. The electrodes were mounted in a customized cell consisting of two parts forming a poly(methyl methacrylate) box, as reported in the literature.^14^ The modified sensor was located in a slot created in the bottom part of the cell, while three screws allowed fixing the top part, where a cylindrical well housed both the illumination system and the solution. The cell volume was 1 mL. An O-ring controlled the tightness at the electrode surface. The geometric area of the electrode was 0.09 cm^2^.

Photocurrent measurements were performed amperometrically at −0.3 V *vs* Ag/AgCl, under illumination with a commercial white light-emitting diode (LED) (5 mm white LED, Conrad Electronics SE, Germany) emitting in the visible range with maxima at 470 nm and intensity in the mW cm^–2^ order. For UV-light illumination, a Camag UV lamp was used, with an emission peak at a wavelength of 366 nm. Open-circuit potential (OCP) measurements were referred to the Ag/AgCl wire pseudo-reference electrode at room temperature (25 °C), in the dark and under illumination.

Cyclic voltammetry (CV) measurements were performed at the scan rate of 0.025 V s^–1^ in the potential range from −0.5 to +0.8 V, unless otherwise stated. Light-chopped linear sweep voltammetry (LSV) measurements were performed in the potential range from +0.1 to −0.4 V at the scan rate of 0.25 mV s^–1^, with a 1 min light off and a 20–30 s light on cycle. Electrochemical impedance spectroscopy (EIS) measurements were performed with a sinusoidal voltage of 0.01 V amplitude, at an OCP value (*vs* the Ag/AgCl pseudo-reference electrode) in the frequency range from 100 kHz to 10 mHz. The EIS spectra were plotted as complex plane diagrams (Nyquist plots). EIS and CV measurements were performed in 1 mL of 0.1 mol dm^–3^ KCl or 5 mmol dm^–3^ Fe(CN)_6_^3–/4–^ solution prepared in 0.1 mol dm^–3^ KCl.

### Calibration Plot

The calibration plot was fitted by nonlinear regression to the four-parameter logistic (4-PL) using Origin Pro 2022 software (Origin Lab Corporation). The limit of detection (LOD) value was evaluated considering the average response of the blank plus three times the standard deviation, whereas the limit of quantification (LOQ) was estimated considering the average response of the blank plus 10 times the standard deviation. The values obtained were converted into moles per liter by fitting the data to the calibration function.

### Singlet Oxygen Determination Using 1,5-Dihydroxynaphthalene (DHN)

The effective capability of the L/TiO_2_/ITO electrode to produce singlet oxygen was evaluated spectrophotometrically (transmittance measurement with a Jasco V-670 spectrophotometer) using 1,5-dihydroxynaphthalene (DHN) as an indirect ^1^O_2_ reporter and by employing a slight modification of previously reported methods.^19,20^ Briefly, a TiO_2_/ITO electrode and a coated L/TiO_2_/ITO electrode, both with a functionalized surface of 1.5 cm × 1.5 cm (2.25 cm^2^), were dipped in 3 mL of a 0.33 mmol dm^–3^ solution of DHN in a mixture of H_2_O and D_2_O in the same proportion with 10% v/v methanol. The samples were irradiated (LED lamp, 30 W, λ c.ca 430 nm) for a total time of 20 minutes, and ultraviolet–visible (UV–vis) spectra were acquired at regular intervals of irradiation time of 5 min. The rate of the photooxidation process of DHN at the electrode surface was obtained by following a decrease in the DHN absorption band (centered at c.ca 297 nm) and the simultaneous increase of the 5-hydroxy-1,4-naphthalendione absorption band (centered at c.ca 427 nm), as described in detail in the Supporting Information (Scheme S3).

### Spectroscopic and NMR Measurements

The Raman spectra have been measured with a Bruker FT-Raman MultiRAM spectrometer (Bruker Optics) equipped with a neodymium-doped yttrium aluminum garnet (Nd–YAG) laser emitting at 1064 nm as the excitation source.

The spectra of L have been recorded in a 0.05 mol dm^–3^ aqueous solution using a quartz cuvette with a 1 cm optical path length and on the L/TiO_2_/ITO substrate.

The NMR spectra were acquired on an Avance III-HD spectrometer (Bruker Biospin) operating at 900 MHz proton Larmor frequency (21 T magnet, Bruker Biospin), equipped with a triple-resonance cryo-cooled probe head. For performing NMR experiments, the powder of titania nanocrystals, detached by scrubbing from the electrode surface, was used. The powder was then resuspended in a 0.05 mol dm^–3^ L aqueous solution and incubated overnight. To the sample thus obtained was added 10% deuterium oxide and kept in suspension within the active volume of the coil by confining it with low-melting agarose. This procedure has been established to keep cells in suspension in bioreactors.^21,22^

### Computational Details

The structural and spectroscopic properties of the ruthenium complex have been carried out within the density-functional theory (DFT) framework with the Gaussian 09 suite of programs^23,24^ using the PBE0 exchange and correlation functional and the LANL2DZ basis set.

The adsorption geometry of the ruthenium complex on the anatase (101) surface has been modeled by performing calculations with the xTB^25,26^ module of the CP2K package.^27,28^

## Results and Discussion

### Electrochemical Characterization

**Figure 1:**
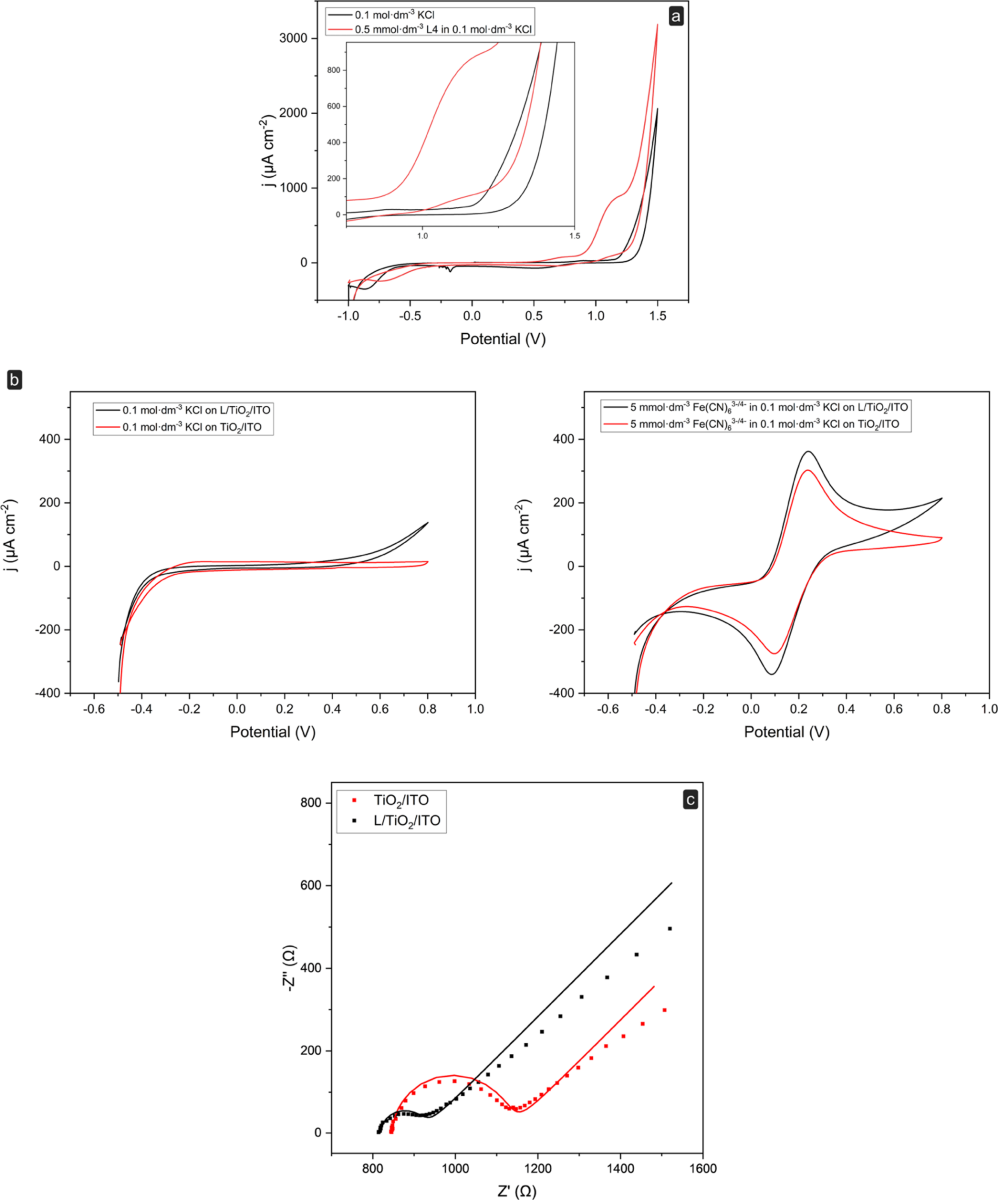
Electrochemical characterization of the Ru(II) complex. (a) Cyclic voltammetry of the 0.5 mmol dm^–3^ Ru(II) complex at a carbon screen-printed electrochemical cell in the presence of 0.1 mol dm^–3^ KCl; potential range: −1 to +1.50 V *vs* Ag/AgCl; scan rate: 0.025 V s^–1^. Inset: expansion of the voltammogram to better visualize the cathodic peak of the Ru(III/II) couple. (b) Cyclic voltammetry scan recorded at the TiO_2_/ITO electrode and the L/TiO_2_/ITO electrode, respectively, in 0.1 mol dm^–3^ KCl (left) and in 0.1 mol dm^–3^ KCl containing 5 mmol dm^–3^ Fe(CN)_6_^3–/4–^ (right). The potential was scanned with a scan rate of 0.025 V s^–1^, from −0.5 to +0.8 V *vs* Ag/AgCl. (c) Nyquist plots recorded in 0.1 mol dm^–3^ KCl containing 5 mmol dm^–3^ Fe(CN)_6_^3–/4–^, at the OCP; experimental data are expressed as points, while lines represent the obtained equivalent circuit fitting.

The electrochemical profile of L in an aqueous solution was obtained using a commercial carbon screen-printed electrochemical cell (SPEC). Figure 1a shows the cyclic voltammetry for a 0.5 mmol dm^–3^ L solution in 0.1 mol dm^–3^ KCl at the SPEC. An oxidation peak was observed in the voltammogram at +1.12 V *vs* Ag/AgCl. This peak is not visible in the scan of the supporting electrolyte solution (0.1 mol dm^–3^ KCl) and can be assigned to the Ru^3+^/Ru^2+^ couple. By contrast, the reduction wave is barely noticeable at around +0.96 V *vs* Ag/AgCl, suggesting that the complex is not quantitatively regenerated on the time scale of the cyclic voltammogram.

The electrochemical, electrical, and structural properties of the L-decorated TiO_2_/ITO electrode (L/TiO_2_/ITO) were studied by CV and by electrochemical impedance spectroscopy. The corresponding CV scans and Nyquist plots are shown in Figure 1b,c, respectively. CV measurements were carried out for the bare TiO_2_/ITO and the modified L/TiO_2_/ITO electrodes in 0.1 mol dm^–3^ KCl (Figure 1b, left panel). The two voltammograms show a large potential range where the current is only capacitive (between −0.4 and +0.2 V), and no faradic phenomena are observed due to the presence of the layer of the photosensitizer at the L/TiO_2_/ITO electrode. Measurements were also performed in the presence of 5 mmol dm^–3^ Fe(CN)_6_^3–/4–^ redox probe in 0.1 mol dm^–3^ KCl (Figure 1b, right panel). The electrochemical behavior of this redox probe is known to be more sensitive to the chemistry and structure of an electrode material surface rather than its electronic density of states.^29^ Both electrodes show a couple of reversible redox peaks even though slightly higher peak currents were observed for the L/TiO_2_/ITO (Table S1). This is consistent with a slight increase in the electroactive surface area due to improved wettability of the surface^30^ after the modification with the photosensitizer and a local increase of the Fe(CN)_6_^3–/4–^ concentration near the electrode surface. Indeed, the L/TiO_2_/ITO electrode may carry positive charges on its surface due to the presence of the highly charged polyazamacrocyclic moieties of L. As reported in Scheme S1, L is a highly charged Ru(II) complex due to the high number of easily protonable nitrogen groups on the two distinct cyclen moieties. At neutral pH (Figure S3), the complex is mostly present as tetra-protonated species [H_4_L]^6+^, in which each macrocyclic unit is in its diprotonated form. The compound is also able to undergo two further protonation steps, but this occurs only in stronger acidic conditions (below pH 4). As reported in Table S2, the first two protonation constants are slightly lower if compared to the first protonation constant of the cyclen unit (log *K* 11.27^31^), as expected due to the presence of the positively charged Ru(II) center in L. On the other hand, these two values (log *K* 11.02 and 10.15, respectively) are similar to each other, indicating that the two H^+^ ions in the diprotonated species of the compound are likely placed on two distinct macrocyclic units. Afterward, analogous to the free cyclen unit, the insertion of a second H^+^ ion on an already protonated cyclen moiety of L is responsible for a significant decrease in the log *K* values corresponding to the third and fourth protonation steps, of 8.53 and 7.59, respectively. The protonation degree of the cyclen units as a function of pH and the acid–base properties of the ruthenium compound were also previously investigated by means of potentiometric measurements.^17^ In conclusion, these positively charged moieties will attract anionic complexes, leading to a local increase of the Fe(CN)_6_^3–/4–^ concentration near the electrode surface, contributing to the increased peak current values at the L/TiO_2_/ITO electrode.

Finally, the impedimetric analysis reported as a Nyquist plot in Figure 1c indicates a reduced charge transfer resistance (*R*_ct_) at the TiO_2_–solution interface after the deposition of the ruthenium(II) complex. In particular, the *R*_ct_ values were found to be 280 ± 14 and 99 ± 11 Ω for the TiO_2_/ITO and L/TiO_2_/ITO platforms, respectively.

### Spectroscopic and Computational Analyses

**Figure 2:**
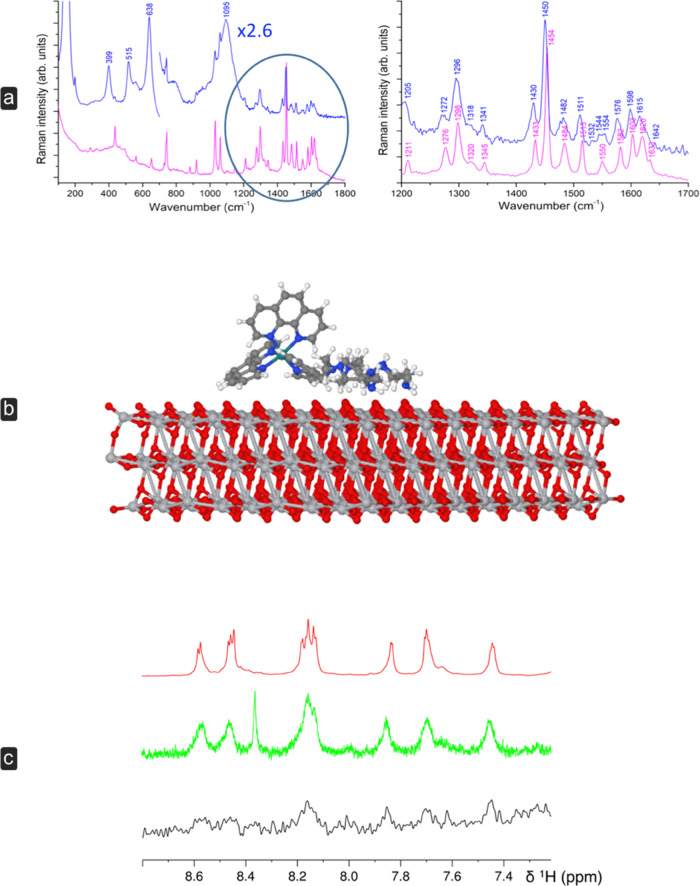
(a) Left: Raman spectra of a 0.05 mol dm^–3^ aqueous solution of the ruthenium complex (magenta) and of the sample adsorbed on the TiO_2_/ITO substrate (blue). Right: Expanded view of the highlighted region. Acquisition settings: 2 cm^–1^ resolution, 450 mW (adsorbed complex), 130 mW (complex solution), incident power, 5000 acquisition scans. (b) xTB geometry optimization results. (c) ^1^H NMR response of the L4 complex upon interaction with the titanium(IV) dioxide particles. Top trace: free complex; middle trace: complex with titanium(IV) dioxide suspended in agarose gel; bottom trace: ^1^H STD response with irradiation at 2.3 ppm.

The Raman spectra of L in solution and L adsorbed on the TiO_2_/ITO electrode (L/TiO_2_/ITO) are compared in Figure 2a. The spectrum of L/TiO_2_/ITO is dominated by the strong peaks at 399, 515, and 638 cm^–1^ due to anatase-TiO_2_ and by the ITO broad band at 1095 cm^–1^. The Raman signals of L are better observed in the 1200–1650 cm^–1^ spectral region, where the strongest peaks of L are located and the TiO_2_/ITO contribution is negligible. In this region, three groups of bands can be identified, the tentative assignment of which has been carried out with the help of DFT calculations on the free complex. The first region, located around 1600 cm^–1^, is characterized by stretching vibrations mainly localized on phenanthroline and bisphenyl ring moieties. The second region contains the intense bands at 1454 cm^–1^, assigned to phenanthroline, and at 1434 cm^–1^ due to both bisphenyl and poliaminic macrocycle vibrations, as well as the third region around 1300 cm^–1^.

As a general trend, most of the Raman bands of L are downshifted by a few wavenumbers on the adsorbed complex (L/TiO_2_/ITO) with respect to the free complex in solution. This effect is more pronounced in the high-frequency region, where larger shifts are observed for the 1620, 1603, and 1582 cm^–1^ bands that move down to 1615, 1598, and 1576 cm^–1^, respectively. The most intense band in the L solution spectrum (at 1454 cm^–1^) is observed at 1450 cm^–1^ in the adsorbed sample (L/TiO_2_/ITO). The presence of such reduced band shifts suggests that the complex molecule is physisorbed on the electrode surface.

Splitting of some Raman bands is also observed moving from the solution to the L/TiO_2_/ITO (solid sample), as in the case of the bands at 1550 and 1484 cm^–1^ that give rise to doublets at 1554, 1544 cm^–1^ and at 1482, 1477 cm^–1^, respectively. The same trend is observed around 1300 cm^–1^, where the 1276 and 1299 cm^–1^ bands split into 1270, 1275 cm^–1^ and 1295, 1298 cm^–1^ doublets, respectively. The appearance of band splitting can be related to a decrease in molecular symmetry induced by the physisorption process to the substrate surface. Finally, both phenanthroline and bisphenyl vibrations are affected by the adsorption process, other than that of the poliaminic macrocycle, suggesting that the whole molecule can slightly interact with the surface.

The UV–vis absorption spectra of L in aqueous media and once adsorbed onto the ITO/TiO_2_ electrode have also been performed (Figure S4). The absorption of L in an aqueous solution (black line) features a broad band centered at about 450 nm, which stems from a metal-to-ligand dπ–π* charge transfer (MLCT) plus a more intense absorption ranging from 260 and 290 nm, which can be attributed to the ligand π–π* transitions of the phenanthroline and bipyridine units. Following adsorption onto the electrode (red line), the maximum of the ligand centered bands of the ruthenium compound turns out to be markedly red-shifted (up to c.ca 80–100 nm), whereas the MLCT band was found to be considerably broadened, indicating the effective interaction of the metal complex with the TiO_2_/ITO electrode. However, it can be evidenced that the MLCT band of L undergoes a considerably lower red-shift effect if compared to the ligand centered transitions, retaining a remarkable absorption within the range of wavelengths 430–480 nm. This would confirm a good match between the absorption spectrum of the dye once adsorbed on the TiO_2_/ITO electrode and the excitation light employed for the photoelectrochemical measurements (LED emitting light with a maximum at 470 nm).

Photographs showing the bare and the L-modified TiO_2_/ITO electrode are reported in Figure S5.

The interaction of the complex with the surface was also probed through saturation-transfer-difference ^1^H NMR spectroscopy,^32^ including spoil, T2-filter, and excitation sculpting to suppress the water signal. The sample was kept in suspension within the active volume of the coil through an agarose gel. The STD was achieved through saturation of the titanium(IV) dioxide terminal −OH resonance at 2.3 ppm.^33^

The response of the molecule in the aromatic region is apparent (shown in Figure 2b), whereas no detectable response could be observed in the aliphatic region because of the strong overlap of the signals from the agarose. Therefore, this is not conclusive evidence about the pose of the complex with respect to the surface. Thus, to further gain insights into the adsorption of the ruthenium complex on the anatase surface, xTB calculations have been performed. xTB geometry optimization results, reported in Figure 2c, show that the interaction with the anatase (101) surface mainly involves the macrocycle arms of the ruthenium complex. However, one of the phenanthroline ligands is close to the surface. Although more accurate studies and calculations are required to provide a definitive description of the interaction between the ruthenium complex and the surface of titanium dioxide, these data are compatible with the spectroscopic results that suggest a physisorption of L on the TiO_2_ surface.

### Photoelectrochemical Behavior

**Figure 3:**
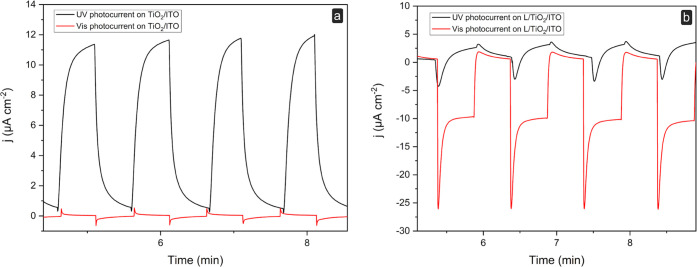
Evaluation of the photoelectrochemical behavior of the L/TiO_2_/ITO electrode: j–t curves at **(a)** bare TiO_2_/ITO and **(b)** L/TiO_2_/ITO electrodes, in 0.1 mol·dm^–3^ KCl in dark and at the irradiation with UV (i) and 470 nm (ii) light. Biased potential of 0 V *vs.* Ag/AgCl.

**Figure 4:**
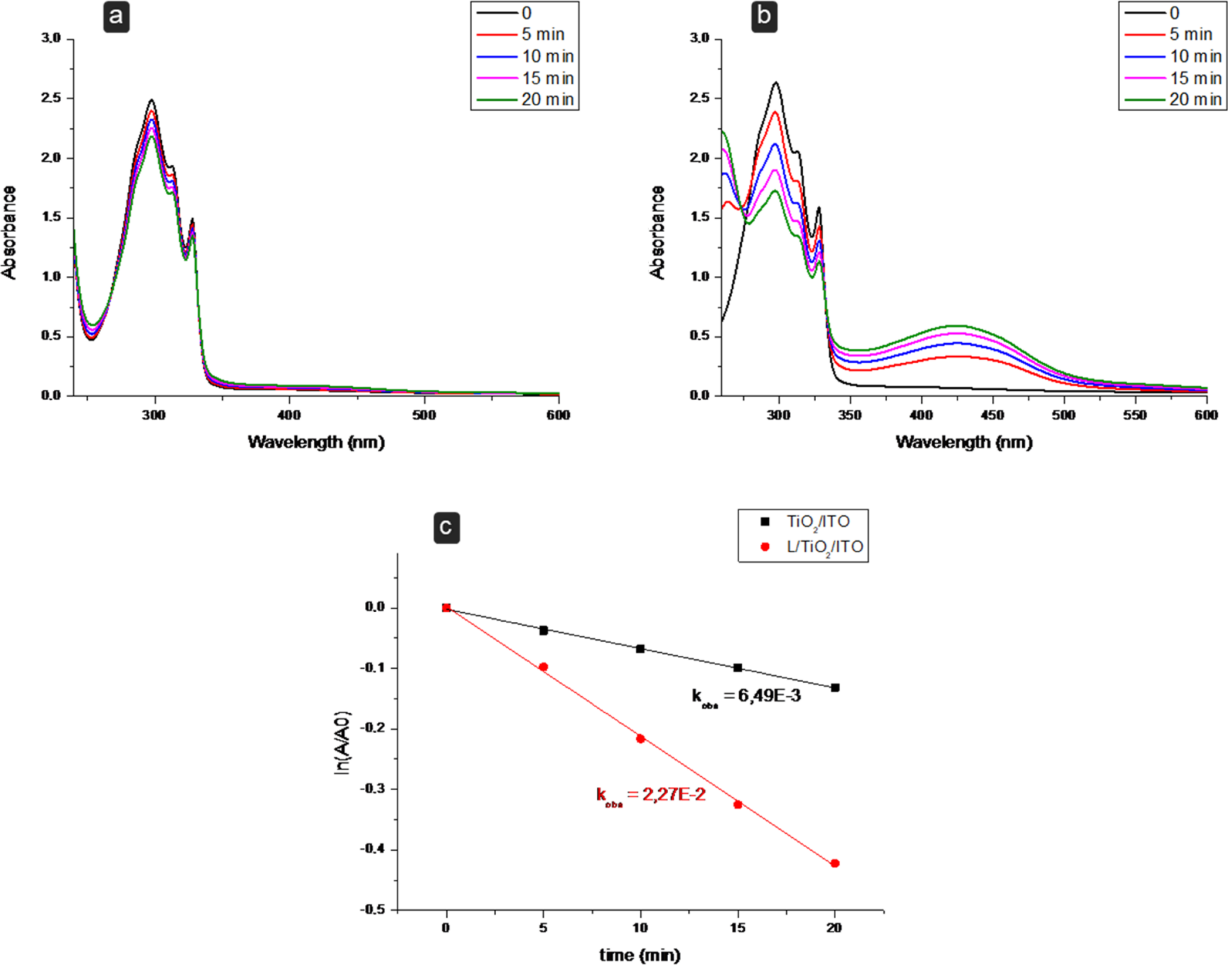
Evaluation of singlet oxygen production using 1,5-dihydroxynaphthalene (DHN). Absorption spectra of aqueous solutions of DHN irradiated with LED light (λ > 430 nm) for different times in the presence of the (a) TiO_2_/ITO electrode with a coated surface of 1.5 cm × 1.5 cm (2.25 cm^2^) and (b) L/TiO_2_/ITO electrode with a functionalized surface of 1.5 cm × 1.5 cm (2.25 cm^2^). (c) Semilogarithmic plots of ln(*A_t_*/*A*_o_) plotted as a function of the irradiation time. Black square, TiO_2_/ITO electrode surface (2.25 cm^2^); red points, L/TiO_2_/ITO electrode surface 2.25 cm^2^. ([DHN] = 3.3·10^–4^ mol dm^–3^).

The photoelectrochemical properties of the L/TiO_2_/ITO electrode were studied by performing photocurrent measurements under UV-light illumination as well as under visible-light illumination. Figure 3 reports the photocurrent responses of both bare TiO_2_/ITO and L-coated TiO_2_/ITO electrodes, under illumination with a UV lamp (366 nm) or a visible LED light (470 nm) at the bias voltage of 0 V in 0.1 mol dm^–3^ KCl. This potential value was chosen for these preliminary experiments since, as shown in Figure 1b, faradic phenomena are absent. The *j*–*t* curves appear anodic for the bare TiO_2_/ITO under UV and visible-light illumination, as expected for an n-type semiconductor such as nanostructured TiO_2_. In addition, at the bare TiO_2_/ITO working electrodes, the photocurrent density is higher under UV illumination than under visible-light illumination (almost negligible). The behavior is different at the L/TiO_2_/ITO because a cathodic photocurrent is always observed at 0 V. In this case, a higher photocurrent density was observed under visible-light illumination than under UV illumination. This behavior is explained by the *in situ* production of singlet oxygen by the ruthenium(II) complex under visible-light illumination (φ_Δ_ = 0.38 ± 0.08, in air-saturated CH_3_CN solution).^10^ Singlet oxygen triggers the appearance of a cathodic photocurrent due to its reduction at the electrode surface. Figure 3b shows the photocurrent signal for a series of four illumination cycles. Good intraelectrode reproducibility was observed (RSD% = 4) by analyzing the photocurrent of singlet oxygen reduction at 0 V (*n* = 4). The *in situ* production of ^1^O_2_ at the L/TiO_2_/ITO surface was evaluated by means of UV–visible spectroscopy using 1,5-dihydroxynaphthalene (DHN) as an indirect reporter for ^1^O_2_. DHN is selectively and quantitatively oxidized, in the presence of ^1^O_2_, to 5-hydroxy-1,4-naphthalendione (juglone)^1^ (see eq 1 and Scheme S3, SI). The generation of ^1^O_2_ can be indeed easily evaluated by monitoring the decrease of the DHN absorption band, centered at 297 nm, and the increase of the band associated with juglone, at 427 nm. Figure 4a,b reports the UV–vis curves obtained for the TiO_2_/ITO and the L/TiO_2_/ITO, subjected to increasing irradiation times. As shown in Figure 4a, irradiation of the bare TiO_2_/ITO electrode results in only a modest change at the DHN absorption band. By contrast, light activation at the L/TiO_2_/ITO determines a strong decrease of the DHN absorption band, along with the simultaneous and progressive increase of the juglone absorption band (Figure 4b). In particular, the rate constant for the DHN photooxidation process (*k*_obs_) increases from 0.0065 ± 0.0001 (min^–1^) for the TiO_2_/ITO electrode to 0.0227 ± 0.0006 (min^–1^) for L/TiO_2_/ITO (Figure 4c). Further details on the determination of the rate constants are reported in the SI. These findings clearly demonstrate the production of ^1^O_2_ at L/TiO_2_/ITO under these experimental conditions (Figure 4b).

### Analysis of Electrochemically Reversible Phenolic Compounds and Bioanalytical Applications

The sensing approach is based on the *in situ* and light-driven production of the reactive ^1^O_2_. Since ^1^O_2_ is a strong oxidant, it can be reduced at the electrode surface when a proper cathodic potential is applied. For this reason, a cathodic current is observed. However, ^1^O_2_ can also chemically react with oxidizable analytes. As an example, ascorbic acid (AA) was tested. AA is a known scavenger molecule of ^1^O_2_ and other reactive oxygen species, and it forms an electrochemical irreversible redox couple with dehydroascorbic acid (DHAA), its oxidation product.^34^ When added to the solution, a decrease in the cathodic photocurrent value was observed (Figure S6). This decrease of the photocurrent confirms that ^1^O_2_, formed at the electrode surface, reacts with AA, and thus, it is less available for electrochemical reduction at the electrode surface.

In contrast, as very recently reported in the literature,^7^^1^O_2_ can also be used to sense electrochemically reversible phenolic compounds, obtaining an increase of the photocurrent. It is known that the hydroxyl moieties’ arrangement on phenol compounds determines their different electrochemical behaviors. Some phenols, such as hydroquinone (HQ), are characterized by reversible electrochemistry involving two electrons and two protons. HQ can be oxidized to benzoquinone (BQ) by photogenerated ^1^O_2_. When a proper negative potential is applied at the electrode surface, BQ can be reduced back to HQ. Such a light-actuated redox cycle produced a photocurrent dependent on the concentration of this phenol in solution (Scheme 1).

**Figure 5:**
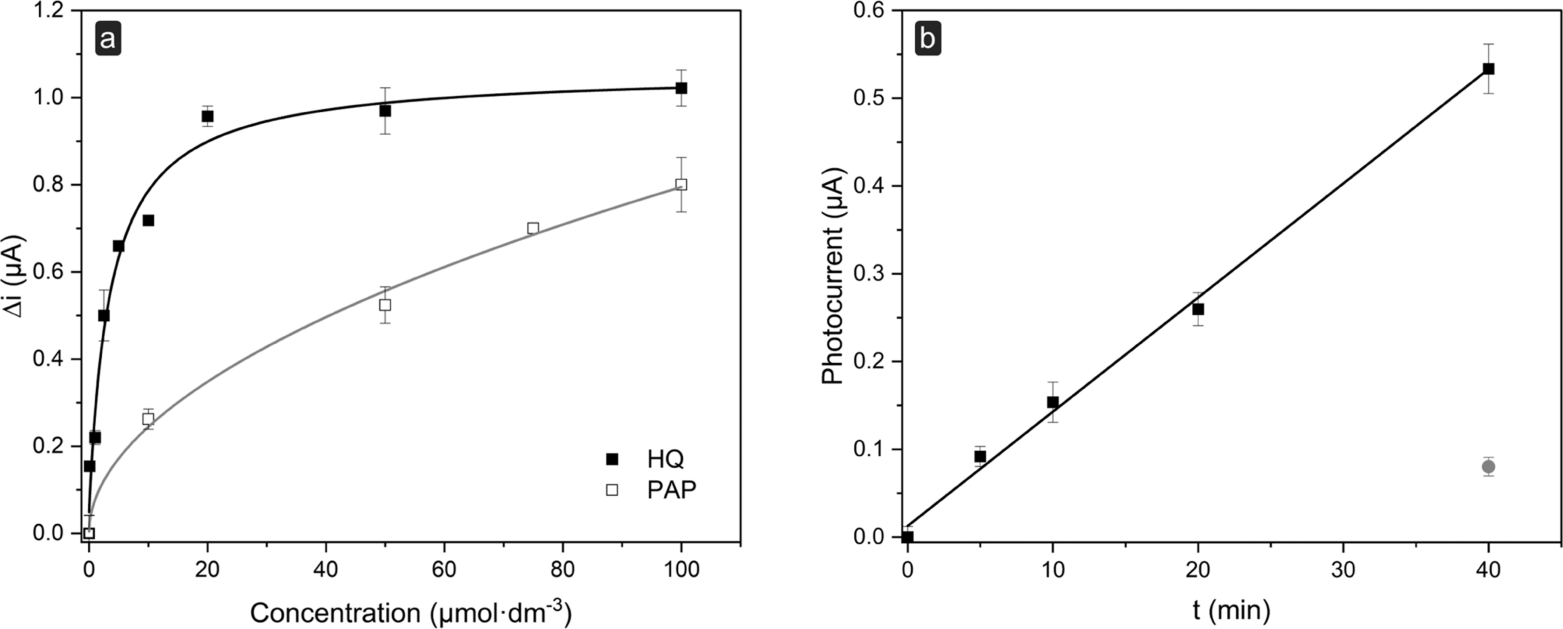
(a) Calibration plots for HQ and PAP at −0.3 V (*vs* Ag/AgCl) in 0.1 mol dm^–3^ glycine buffer pH 7.0, containing 0.1 mol dm^–3^ KCl. Data for the calibration curve are presented as mean (±s.d.) of at least three consecutive measurements. (b) Current trend over time as a result of the enzymatic reaction between pAPP and AP and the subsequent reduction at the electrode surface of the enzymatically produced PAP. The red dot represents the photocurrent value after 40 min in the absence of AP. Measurements were performed in 0.1 mol dm^–3^ glycine buffer pH 9.0, containing 0.1 mol dm^–3^ KCl.

Indeed, the sensitivity of the electrode is highly influenced by the applied potential. Thus, its effect was investigated over a potential range from 0 to −0.4 V in a solution containing 50 μmol dm^–3^ HQ. The photocurrent and the background current increase as the applied potential shifts toward more negative values (Figure S7a) due to the presence of other reactions, such as the reduction of oxygen. The value of −0.3 V *vs* Ag/AgCl was chosen as the optimal working potential. Furthermore, the pH-dependent speciation of phenols is pivotal when studying their photooxidation kinetics. However, as reported in the literature,^35,36^ the oxidation rate increases along with the pH (Figure S7b). Considering the fact that protons participate in the electrochemical reaction of phenols, a buffered solution was used, and hence, a glycine buffer at pH = 7.0 was chosen for further experiments. To explore the applicability of the proposed sensing approach, HQ was measured at L/TiO_2_/ITO under visible-light illumination. The calibration plot (Figure 5a) exhibits an increase in the current response, increasing the HQ concentration in the range 0–100 μmol dm^–3^.

The photocurrent response to HQ is linear in the low micromolar concentration range, starting to deviate from linearity at 10 μmol dm^–3^ and leveling off above 50 μmol dm^–3^; this behavior is probably due to a local decrease of reagents at the electrode interface.

The LOD and the LOQ calculated for HQ were 0.3 and 2.1 μmol dm^–3^, respectively. The L/TiO_2_/ITO sensor has shown an LOD value better than or comparable to those of other electrochemical methods for HQ determination, as shown in Table S3.

The precision has been evaluated by repeating the measure of the photocurrent using the L/TiO_2_/ITO electrode for each measurement (i.e., under repeatability conditions). Concentration levels of 10 μmol dm^–3^ have been tested with %RSD of 11% (*n* = 4). Batch-to-batch reproducibility was also assessed, obtaining an RSD% value of 20% (*n* = 3).

Alkaline phosphatase (AP) is one of the most commonly used enzyme labels in immunoenzymatic assays, such as the enzyme-linked immunosorbent assay (ELISA). AP catalyzes the hydrolysis of phosphate esters to give an organic compound and an inorganic phosphate. The hydrolysis reaction is generally followed by spectrophotometry using *p*-nitrophenyl phosphate, by fluorescence with fluorescein phosphate, by chemiluminescence using dioxetane phosphate, and by amperometry with 1-naphthyl phosphate or *p*-aminophenyl phosphate (pAPP). To date, pAPP has been defined as one of the best substrates for ELISA with amperometric detection,^37^ and electrochemical affinity assays using this substrate have been reported.

Thus, the L/TiO_2_/ITO electrode was challenged for the detection of *p*-aminophenol (PAP) as a product of the AP enzymatic hydrolysis of pAPP. PAP is a phenol known to show a quasi-reversible electrochemical behavior.^12^ As expected, an increase in the photocurrent was observed on increasing the PAP concentration in solution. Briefly, PAP is oxidized at the electrode by ^1^O_2_ to form *p*-iminoquinone, which is electrochemically reduced to PAP at the electrode surface. The calibration plot, in the range 0–100 μmol dm^–3^, is reported in Figure 5a. The LOD and the LOQ calculated for PAP were 1.9 and 22 μmol dm^–3^, respectively. Glycine buffer pH 7.0, containing 0.1 mol dm^–3^ KCl, was used for this set of experiments.

The electrode was then challenged for the time-dependent measurement of the AP reaction in the presence of pAPP. This measurement allows the evaluation of the enzyme activity, evaluating the enzymatic reaction between pAPP and AP and the concomitant production of PAP. The pAPP sample solution in the absence of AP was also tested, although just a slight change in the photocurrent measured during 40 min was noted, confirming that the effect of the spontaneous hydrolysis of pAPP is negligible in this time frame (Figure 5b). As known, the measurement of AP activity has to be performed at pH > 9; for this reason, preliminary experiments regarding the optimization of the potential value at this pH value were performed (Figure S8a). Nevertheless, as reported in the literature,^37^ despite not being optimal for PAP measurement (Figure S8b), this pH value can represent a good compromise between AP activity and PAP stability.

The enzyme assay was carried out by preparing a standard solution of pAPP (2.5 nmol dm^–3^). The reaction was started by adding a known amount of enzyme (0.01 U mL^–1^). The enzymatic activity is measured by amperometric detection of the reduction current generated by the chemically oxidized substrate at the L/TiO_2_/ITO electrode illuminated by visible light. As reported in Figure 5b, the current rises with time, with a linear behavior. From these preliminary experiments, we can conclude that the proposed method can be used to evaluate the enzymatic activity and thus can be considered an interesting way to monitor a bioaffinity reaction using AP as the enzymatic label in PEC bioaffinity assays.

## Conclusions

For the first time, we used the ability of a ruthenium(II) polypyridyl complex (L) to generate singlet oxygen for the photoelectrochemical sensing of reversible phenolic compounds. L was deposited on a TiO_2_/ITO electrode. Raman and NMR data as well as xTB calculations suggested the physisorption of L on the TiO_2_ surface. A (photo)electrochemical characterization of the L/TiO_2_/ITO was performed. The production of ^1^O_2_ at the electrode surface was assessed by spectrophotometric measurements. ^1^O_2_ was used to trigger the oxidation of reversible and quasi-reversible phenols, such as hydroquinone, when irradiated. Hydroquinone reacted with ^1^O_2_, producing benzoquinone, which was reduced back at the electrode surface in a redox cycle, generating a detectable photocurrent correlated to its concentration. An LOD of 0.3 μmol dm^–3^ was obtained. Furthermore, harnessing the activity of AP, the L/TiO_2_/ITO electrode was tested for the detection of PAP. Notably, PAP was *in situ* produced from the enzymatic reaction of pAPP with AP on the electrode surface. As a result, a detectable photocurrent was observed. The monitoring of PAP production over time can be used for the assessment of the enzyme activity in PEC bioassays.

## Supporting Information Available

Additional experimental details, materials, and methods, including graphs and photographs of the experimental setup (PDF)
