# Supplementary material for: Characterization of a Ruthenium(II) Complex in Singlet Oxygen-Mediated Photoelectrochemical Sensing
Source: Langmuir. 2022 Dec 27;39(1):679–89. doi: 10.1021/acs.langmuir.2c03042 (PMC9835978; doi:10.1021/acs.langmuir.2c03042)
Supplement: Supplementary file 1 — la2c03042_si_001.pdf [file la2c03042_si_001.pdf]

Supporting information

Characterization of a ruthenium(II) complex in singlet oxygen-mediated photoelectrochemical sensing

Margherita Verrucchi[§,°], *Gina Elena Giacomazzo*[§,°], *Patrick Severin Sfragano*[§,°], *Serena Laschi*[§], *Luca Conti*[§], *Marco Pagliai*[§], *Cristina Gellini*[§], *Marilena Ricci*[§], *Enrico Ravera*[§,#,+], *Barbara Valtancoli*[§], *Claudia Giorgi*[§,*], *Ilaria Palchetti*[§,*]

[§]Dipartimento di Chimica Ugo Schiff, Università degli Studi di Firenze, Via della Lastruccia 3, 50019, Sesto Fiorentino (FI), Italy

[#]CERM, Università degli Studi di Firenze, Via Luigi Sacconi 6, 50019 Sesto Fiorentino (FI), Italy

[+]CIRMMP, Via Luigi Sacconi 6, 50019 Sesto Fiorentino (FI), Italy

* Corresponding Authors:

*Ilaria Palchetti: ilaria.palchetti@unifi.it

*Claudia Giorgi: claudia.giorgi@unifi.it

°*M.V., P.S.S, and G.E.G. contributed equally to this paper.*

KEYWORDS: ruthenium(II) complexes, singlet oxygen, photoelectrochemistry, ascorbic acid, hydroquinone, *p*-aminophenol

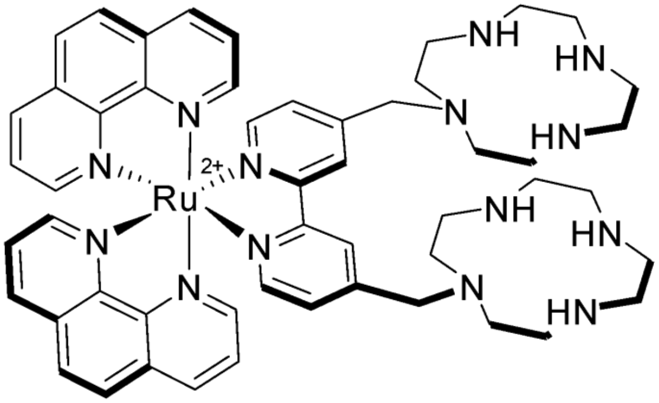

Scheme S1. Molecular structure of the ruthenium(II) complex L.

Synthesis and characterization of L

$Ru(phen)_2Cl_2$ was prepared accordingly to methods described in the literature [1,2]. The synthesis of ligand 4,4'-bis[methylen-(1,4,7,10-tetraazacyclododecane)]-2,2'-bipyridine and of the corresponding ruthenium(II) complex were reported in a previous paper. Briefly, $Ru(phen)_2Cl_2$ was allowed to directly react with an equimolar amount of the bidentate ligand in ethylene glycol, heating the reaction mixture at 160 °C (under microwave irradiation 60 W) for 8 minutes. The ligand L was obtained in good yield (> 60%).

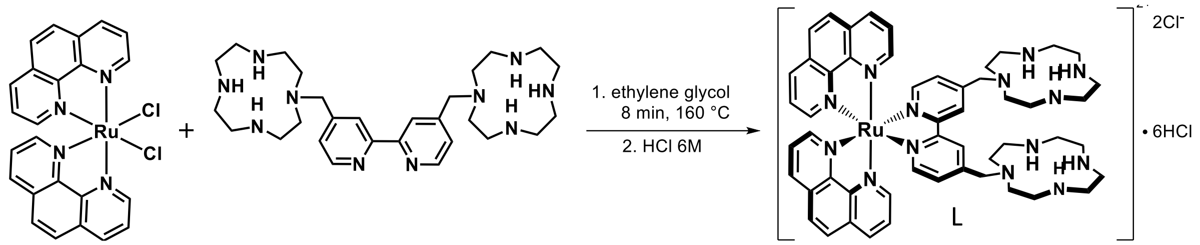

Scheme S2. Synthesis of L.

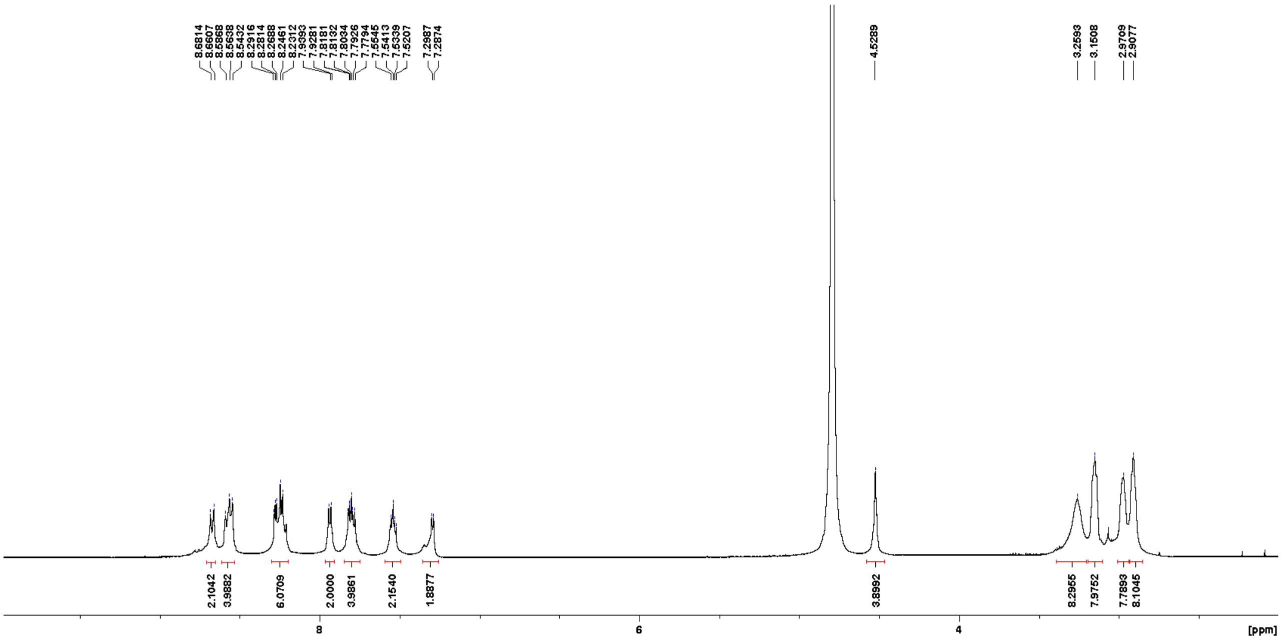

Figure S1. ^{1}H NMR spectra of L (D_2O, pD < 2, 400 MHz): δ(ppm) 8.67 (d, 2H, J = 8.28 Hz), 8.61-8.53 (m, 4H), 8.33-8.2[illegible] (m, 6H), 7.93 (d, 2H, J = 4.48 Hz), 7.84-7.75 (m, 4H), 7.58-7.52 (m, 2H), 7.29 (d, 2H, J = 4.52 Hz), 4.52 (s, 4H, -CH_2), 3.37-3.19 (m, 8H), 3.19-3.09 (m, 8H), 3.04-2.94 (m, 8H), 2.94-2.76 (m, 8H).

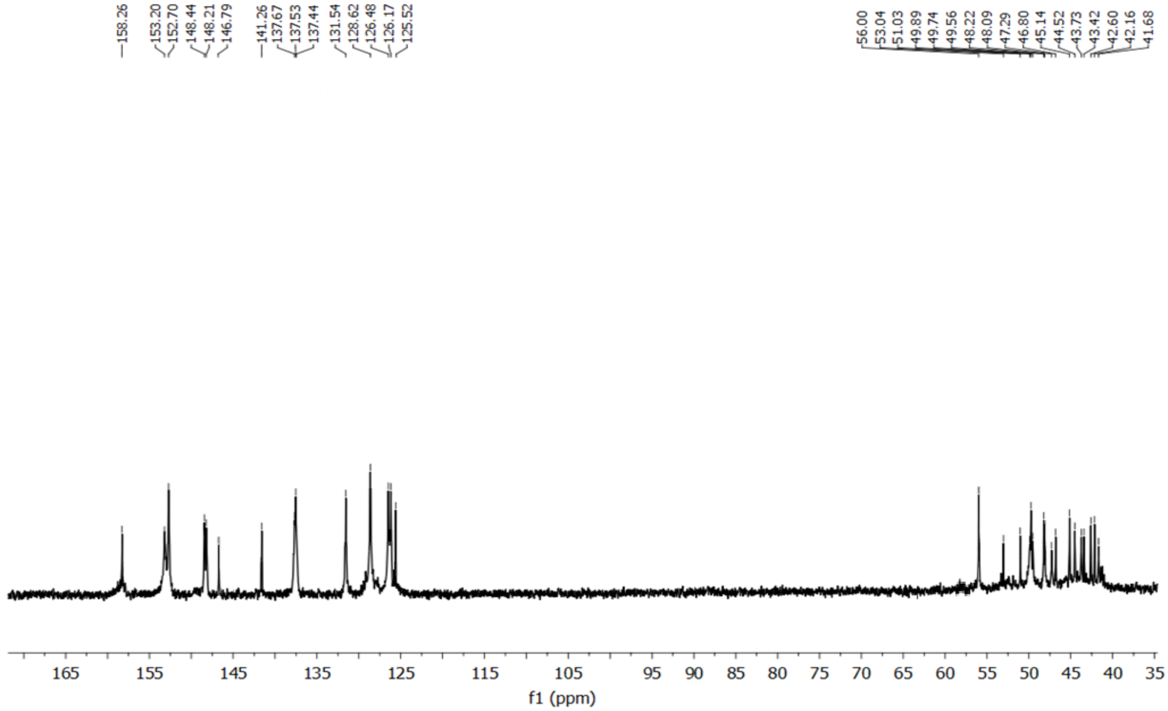

Figure S2. ^{13}C NMR spectra of L (D_2O, pD < 2, 400 MHz): δ(ppm) 158.26; 153.20; 152.70; 148.44; 148.21; 146.79; 141.26; 137.67; 137.53; 137.44; 131.54; 128.62; 126.46; 126.17; 125.52; 56.00; 53.04; 51.03; 49.89; 49.74; 49.56; 48.22; 48.09; 47.29; 46.80; 45.14; 44.52; 43.73; 43.42; 42.60; 42.16; 41.68.

Singlet oxygen determination by 1,5-Dihydroxynaphthalene (DHN)

The capacity of L/TiO_2/ITO electrode to produce singlet oxygen ($^1O_2$) upon photo-activation was studied using 1,5-dihydroxynaphthalene (DHN) as indirect chemical probe for $^1O_2$. DHN is selectively and quantitatively oxidized by $^1O_2$ to give 5-hydroxy-1,4-naphthalenedione (Juglone) according to Scheme S2 and Equation 1:

$$v = kr[^1O_2][DHN] \qquad [1]$$

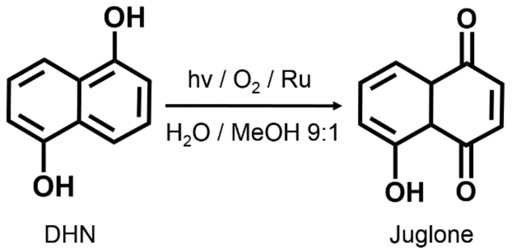

Scheme S3. Photochemical conversion of DHN to Juglone.

The photo-oxidation process is monitored by means of UV-visible spectroscopy following the decrease of the DHN absorption band (λ_{max} 297 nm) with the contemporary increase of Juglone absorption band (λ_{max} 427 nm). Considering the $^1O_2$ concentration constant at the initial stage of reaction, it is possible to apply the steady-state approximation. In these conditions, the DHN consumption coincides with the disappearance rate of $^1O_2$, and the rate of the photo-oxidation process can be written as in Equation 2 using a pseudo-first-order rate constant (k_{obs}). Furthermore, since the absorbance at 297 nm can be univocally attributed to DHN, the rate constant k_{obs} can be obtained as semilogarithmic plot of $\ln(A_{297})_t/(A_{297})_0$ as a function of irradiation time by using Equation 3, where $[DHN]_0$ and $[DHN]_t$ are

the molar concentration of DHN at time ‘zero’ and at a generic time ‘*t*’, while $A(_{297})_0$ and $A(_{297})_t$ are the correspondent absorbance values measured at 297 nm.

$$v = k_{obs} [DHN] \quad [2]$$

and

$$\ln(C_1/C_0) = -k_{obs}\, t \quad [3]$$

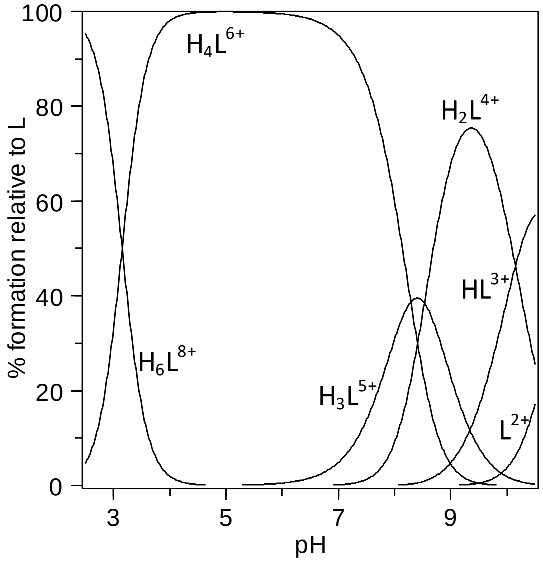

Figure S3. Distribution diagram of the protonated species of L (L = [Ru(phen)$_2$(L')]$^{2+}$).

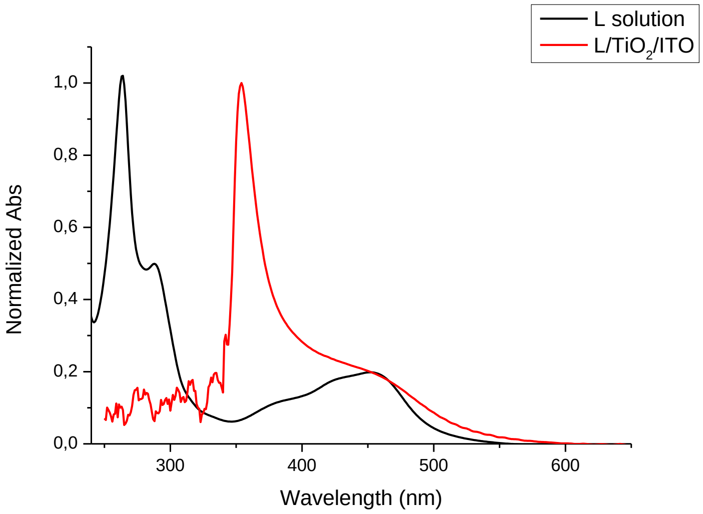

Figure S4. Normalized absorption spectra of an aqueous solution of L (black line) and L/TiO_2/ITO electrode with a coated surface of 1 cm × 2 cm (2 cm^2) (red line).

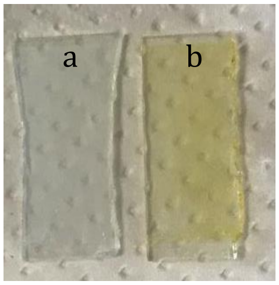

Figure S5. Photograph showing the two electrodes: **(a)** bare TiO_2/ITO and **(b)** L/TiO_2/ITO.

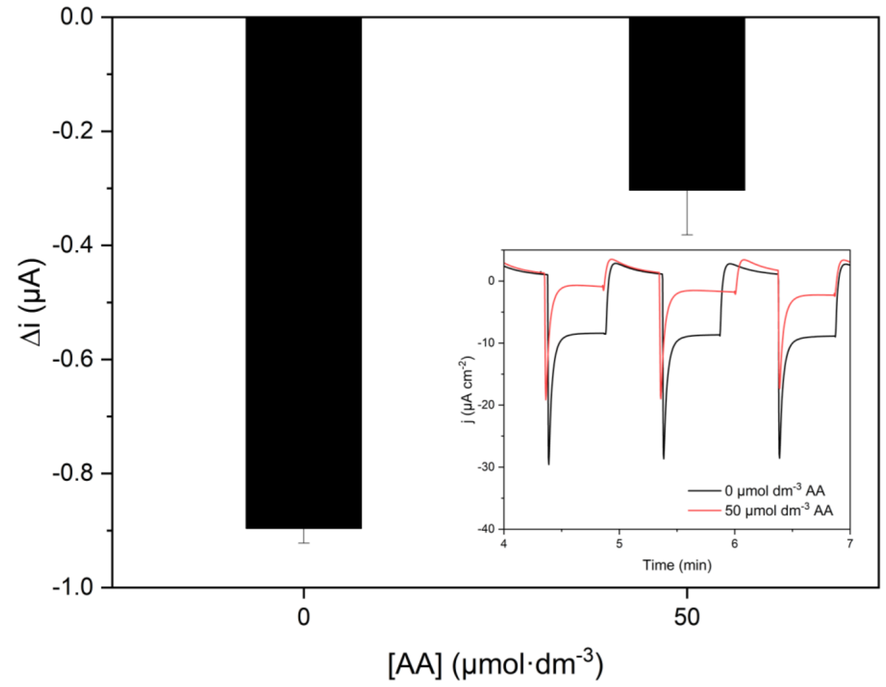

Figure S6. Photocurrent measurements performed in 0.1 mol·dm^{-3} KCl in presence of 0 and 50 μmol·dm^{-3} ascorbic acid upon irradiation with 470 nm light. The inset shows the photocurrent densities when turning on and off the light, in presence and absence of ascorbic acid. Biased potential of 0 V *vs.* Ag/AgCl.

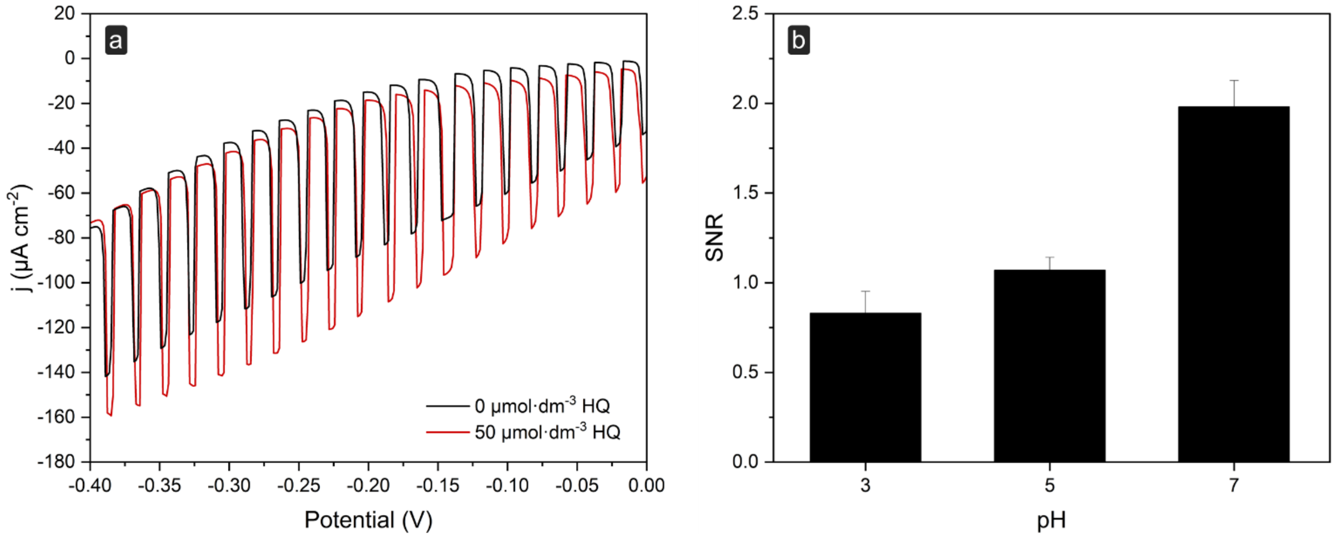

Figure S7. (a) Effect of the electrode potential on HQ determination by linear sweep voltammetry (LSV) scan at L/TiO_2/ITO electrode in the absence and presence of 50 µmol·dm^{-3} HQ in 0.1 mol·dm^{-3} KCl. Scans range from 0 to -0.4 V with a scan rate of 0.25 mV·s^{-1}. 1 min light off, 20 s light on; **(b)** Effect of pH on HQ determination; photocurrent of 100 µmol·dm^{-3} HQ at different pH values; the data are represented as signal-to-noise ratios (SNRs), dividing the photocurrent generated upon irradiation when applying -0.3 V in presence of 100 µmol·dm^{-3} HQ by the response of the blank (0.1 mol·dm^{-3} glycine buffer containing 0.1 mol·dm^{-3} KCl) at pH 3, 5, and 7.

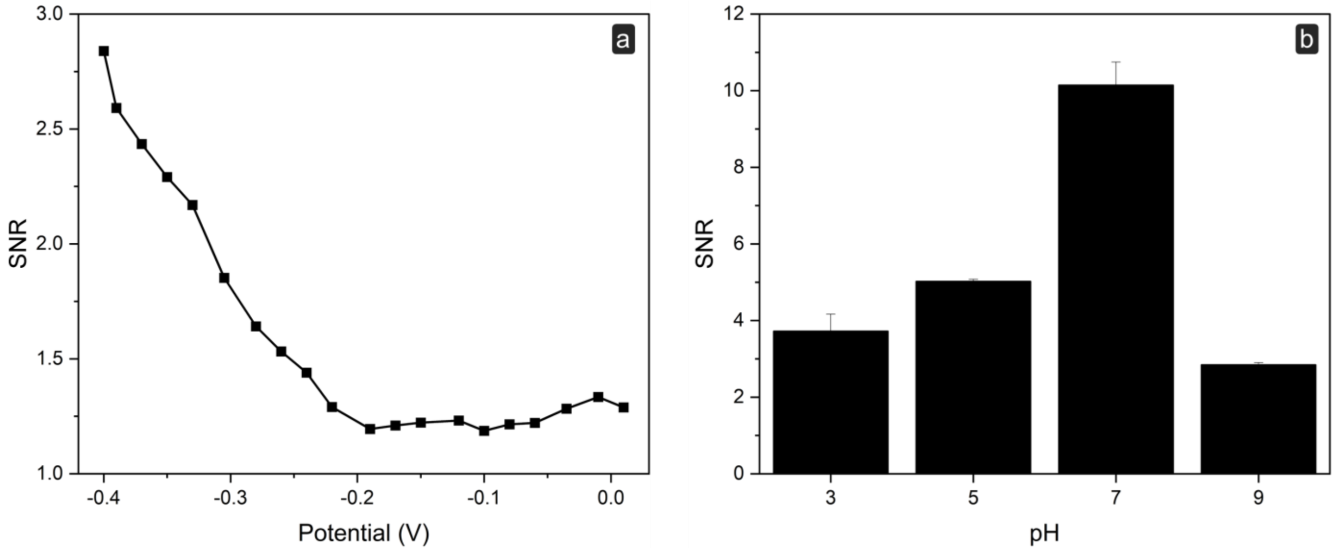

Figure S8. (a) Effect of potential, expressed as signal-to-noise ratio, on PAP determination by linear sweep voltammetry at L/TiO_2/ITO electrode in the absence and presence of 100 μmol·dm^{-3} PAP in 0.1 mol·dm^{-3} glycine buffer at pH 9 containing 0.1 mol·dm^{-3} KCl. Scan ranges from +0.1 to -0.4 V, with a scan rate of 0.25 mV·s^{-1}. 1 min light off, 30 s light on; **(b)** Effect of pH on PAP determination; photocurrent of 100 μmol·dm^{-3} PAP at different pH values; the data are represented as signal-to-noise ratios (SNRs), dividing the photocurrent generated upon irradiation when applying -0.4 V in presence of 100 μmol·dm^{-3} PAP by the response of the blank (0.1 mol·dm^{-3} glycine buffer containing 0.1 mol·dm^{-3} KCl) at pH 3, 5, 7, and 9.

Table S1. Electrochemical parameters obtained from the CV scans recorded at the TiO_2/ITO and electrodes L/TiO_2/ITO for an equimolar solution containing 2.5 mmol·dm^{-3} $Fe(CN)_6^{3-}$ and 2.5 mmol·dm^{-3} $Fe(CN)_6^{4-}$ in 0.1 mol·dm^{-3} KCl. The potential was scanned with a scan rate of 25 mV·s^{-1}.

**Electrode**	**i_{pa} (μA)**	**i_{pc} (μA)**	**ΔE (mV)**	**i_{pa}/i_{pc}**
TiO_2/ITO	23.63	-20.70	140.7	1.14
L/TiO_2/ITO	35.39	-30.47	154.1	1.16

Table S2. Protonation constants of L determined by means of potentiometric measurements in NaCl 0.1 mol·dm^{-3} aqueous solution at 298 K. Values on parentheses are errors on the last significant figure.

Reaction	Log K
$L^{2+} + H^+ = HL^{3+}$	11.02 (3)
$HL^{3+} + H^+ = H_2L^{4+}$	10.15 (4)
$H_2L^{4+} + H^+ = H_3L^{5+}$	8.53 (5)
$H_3L^{5+} + H^+ = H_4L^{6+}$	7.59 (3)
$H_4L^{6+} + 2H^+ = H_6L^{8+}$	7.01 (7)

Table S3. LODs and dynamic ranges of state-of-art methods for HQ and PAP electrochemical determination.

	Electrode	Electroanalytical method	LOD (μmol·dm^{-3})	Concentration range	Ref.
Hydroquinone	CPE modified with 6.0% (m/m) decanoic acid and TBAB-based HDES	Voltammetry	0.77	2.5–3000 μmol·dm^{-3}	3
	Nanometer cobalt and poly-L-glutamic acid modified GCE	Voltammetry	0.497	3.85 -1300 μmol·dm^{-3}	4
	Direct green 6 decorated CPE	Voltammetry	0.11	5-45 μmol·dm^{-3}	5
	SPCE functionalized with carbon spherical shells	PEC	2.7	3 - 23 μmol·dm^{-3}	6
	F64PcZn|TiO_2-coated SPE	PEC	0.026	0.05–2.00 μmol·dm^{-3}	7
	**L/TiO_2/ITO**	PEC	0.3	0-100 μmol·dm^{-3}	This work
p-aminophenol	VMSF/ITO		0.32	0.5-400 μmol·dm^{-3}	8
	GCE	DPV	0.39	0.1-1 μmol·dm^{-3}	9
	TiO_2	FIA-PEC	0.018	0.0125–1.0 μmol·dm^{-3}	10
	G-PANI/CPE		15.68	50-500 μmol·dm^{-3}	11
	Carbon disk	CE-AD	1.4	10-1000 μmol·dm^{-3}	12
	**L/TiO_2/ITO**	PEC	1.9	0.1-100 μmol·dm^{-3}	This work

CPE: carbon paste electrode; TBAB: tetrabutylammonium bromide; HDES: hydrophobic deep eutectic solvents; GCE: glassy carbon electrode; SPCE: screen-printed carbon electrode; PEC: photoelectrochemistry; VMSF/ITO: ITO modified with vertically-ordered mesoporous silica-nanochannel films; GCE: glassy carbon electrode; DPV: differential pulse voltammetry; G-PANI/CPE: graphene-polyaniline modified carbon paste electrode; CE-AD: capillary electrophoresis with amperometric detection.

References

(1) Wachter, E.; Heidary, D. K.; Howerton, B. S.; Parkin, S.; Glazer, E. C. Light-Activated Ruthenium Complexes Photobind DNA and Are Cytotoxic in the Photodynamic Therapy Window. *Chem. Commun.* **2012**, *48* (77), 9649. https://doi.org/10.1039/c2cc33359g.

(2) Conti, L.; Mummolo, L.; Romano, G. M.; Giorgi, C.; Giacomazzo, G. E.; Prodi, L.; Bencini, A. Exploring the Ability of Luminescent Metal Assemblies to Bind and Sense Anionic or Ionizable Analytes A Ru(Phen)2bipy-Based Dizinc Complex for Bisphenol A (BPA) Recognition. *Molecules* **2021**, *26* (3), 527. https://doi.org/10.3390/molecules26030527.

(3) Augusto, K. K. de L.; Piton, G. R.; Gomes-Júnior, P. C.; Longatto, G. P.; de Moraes, F. C.; Fatibello-Filho, O. Enhancing the Electrochemical Sensitivity of Hydroquinone Using a Hydrophobic Deep Eutectic Solvent-Based Carbon Paste Electrode. *Anal. Methods* **2022**, *14* (20), 2003–2013. https://doi.org/10.1039/D2AY00473A.

(4) Huang, B.; Yao, C.; Yang, J.; Du, S.; Lu, X. A Study on the Electrochemical Behavior of Hydroquinone at a Nanometer Cobalt/ <scp>l</Scp> -Glutamate-Modified Electrode. *RSC Adv.* **2020**, *10* (71), 43834–43839. https://doi.org/10.1039/D0RA07222B.

(5) Chetankumar, K.; Kumara Swamy, B. E.; Sharma, S. C.; Hariprasad, S. A. An Efficient Electrochemical Sensing of Hazardous Catechol and Hydroquinone at Direct Green 6 Decorated Carbon Paste Electrode. *Sci. Rep.* **2021**, *11* (1), 15064. https://doi.org/10.1038/s41598-021-93749-w.

(6) Martoni, L. V. L.; Gomes, N. O.; Prado, T. M.; Calegaro, M. L.; Oliveira Jr., O. N.; Machado, S. A. S.; Raymundo-Pereira, P. A. Carbon Spherical Shells in a Flexible Photoelectrochemical Sensor to Determine Hydroquinone in Tap Water. *J. Environ. Chem. Eng.* **2022**, *10* (3), 107556. https://doi.org/10.1016/j.jece.2022.107556.

(7) Neven, L.; Barich, H.; Sleegers, N.; Cánovas, R.; Debruyne, G.; De Wael, K. Development of a Combi-Electrosensor for the Detection of Phenol by Combining Photoelectrochemistry and Square Wave Voltammetry. *Anal. Chim. Acta* **2022**, *1206*, 339732. https://doi.org/10.1016/j.aca.2022.339732.

(8) Liu, X.; Li, H.; Zhou, H.; Liu, J.; Li, L.; Liu, J.; Yan, F.; Luo, T. Direct Electrochemical Detection of 4-Aminophenol in Pharmaceuticals Using ITO Electrodes Modified with Vertically-Ordered Mesoporous Silica-Nanochannel Films. *J. Electroanal. Chem.* **2020**, *878*, 114568. https://doi.org/10.1016/j.jelechem.2020.114568.

(9) de Souza, J. C.; Zanoni, M. V. B.; Oliveira-Brett, A. M. Reprint of "Genotoxic Permanent Hair Dye Precursors p-Aminophenol and p-Toluenediamine Electrochemical Oxidation Mechanisms and Evaluation in Biological Fluids." *J. Electroanal. Chem.* **2020**, *872*, 114529. https://doi.org/10.1016/j.jelechem.2020.114529.

(10) Mendonça, C. D.; Rahemi, V.; Hereijgers, J.; Breugelmans, T.; Machado, S. A. S.; De Wael, K. Integration of a Photoelectrochemical Cell in a Flow System for Quantification of 4-Aminophenol with Titanium Dioxide. *Electrochem. commun.* **2020**, *117*, 106767. https://doi.org/10.1016/j.elecom.2020.106767.

(11) Rattanarat, P.; Suea-Ngam, A.; Ruecha, N.; Siangproh, W.; Henry, C. S.; Srisa-Art, M.; Chailapakul, O. Graphene-Polyaniline Modified Electrochemical Droplet-Based Microfluidic Sensor for High-Throughput Determination of 4-Aminophenol. *Anal. Chim. Acta* **2016**, *925*, 51–60. https://doi.org/10.1016/j.aca.2016.03.010.

(12) Chu, Q.; Jiang, L.; Tian, X.; Ye, J. Rapid Determination of Acetaminophen and P-Aminophenol in Pharmaceutical Formulations Using Miniaturized Capillary Electrophoresis with Amperometric Detection. *Anal. Chim. Acta* **2008**, *606* (2), 246–251. https://doi.org/10.1016/j.aca.2007.11.015.
